# Sustained Long-Term Retention Rates of Abatacept in Combination with Conventional Synthetic Disease-Modifying Antirheumatic Drugs in Elderly Patients with Rheumatoid Arthritis

**DOI:** 10.3390/medicina57090914

**Published:** 2021-08-31

**Authors:** Shuzo Sato, Haruki Matsumoto, Jumpei Temmoku, Yuya Fujita, Naoki Matsuoka, Makiko Yashiro-Furuya, Tomoyuki Asano, Eiji Suzuki, Hiroshi Watanabe, Takashi Kanno, Kiyoshi Migita

**Affiliations:** 1 Department of Rheumatology, Fukushima Medical University School of Medicine, 1 Hikarigaoka, Fukushima 960-1295, Fukushima, Japan; haruki91@fmu.ac.jp (H.M.); temmoku@fmu.ac.jp (J.T.); fujita31@fmu.ac.jp (Y.F.); naoki-11@fmu.ac.jp (N.M.); myashiro@fmu.ac.jp (M.Y.-F.); asanovic@fmu.ac.jp (T.A.); chiehiro@fmu.ac.jp (H.W.); migita@fmu.ac.jp (K.M.); 2 Department of Rheumatology, Ohta Nishinouchi Hospital, Koriyama 963-8558, Fukushima, Japan; azsuzuki@ohta-hp.or.jp (E.S.); kanno-t@ohta-hp.or.jp (T.K.)

**Keywords:** abatacept, DMARD, elderly, rheumatoid arthritis, tacrolimus

## Abstract

*Background and Objectives*: Treatment for elderly (aged ≥75 years) patients with rheumatoid arthritis (RA) is important because they usually have several complications and organ dysfunction and are more susceptible to drug-related adverse events. Abatacept (ABT) treatment is relatively safe in elderly RA patients; however, the real-world data of efficacy and long-term retention of ABT is sparse in such patients. This study aimed to investigate the clinical efficacy and long-term retention rates of ABT in elderly Japanese RA patients. *Materials and Methods*: This 10-year retrospective observational cohort study was performed in two centers in Fukushima, Japan. We reviewed the clinical features of elderly RA patients who received ABT and investigated the differences in retention rates with concomitant administration of conventional synthetic disease-modifying antirheumatic drugs (csDMARDs). *Results:* The clinical characteristics of younger (<75 years old, 39 cases) and elderly (≥75 years old, 20 cases) RA patients were generally similar. Although the efficacy was also similar, the concomitant administration of csDMARDs with ABT differed between the two groups. Younger patients significantly decreased methotrexate (MTX) administration than elderly patients (*p* < 0.01), and elderly patients significantly received tacrolimus (TAC) (*p* < 0.01) or salazosulfapyridine (SASP; *p* = 0.01) than younger patients. The overall retention and infection-free survival rates were similar between the two groups. *Conclusion:* Elderly RA patients showed sustained retention rates compared to younger RA patients. TAC and SASP can help to maintain sustained retention rates in elderly RA patients.

## 1. Introduction

The population of elderly patients with rheumatoid arthritis (RA) is increasing [1], and treatment, especially of those aged ≥75 years, is a critical issue because these patients may have complications and organ dysfunction and be more susceptive to adverse drug events [2,3,4]. Treatment with abatacept (ABT), a biologic agent that suppresses T cells by blocking co-stimulation signals, is safer in elderly RA patients compared with other biologics [3,5,6]. Furthermore, recent reports have shown that ABT retention is relatively higher than other biologics [7,8]. For instance, Ebina et al. have reported that ABT retention was higher than other biologics at 35 months of treatment in the Japanese cohort (ANSWER study) [7]. However, data on the long-term retention and efficacy of ABT in elderly RA patients, especially those aged ≥75 years, are sparse. Furthermore, the desirable combination therapy with ABT and conventional synthetic (cs) disease-modifying antirheumatic drugs (DMARDs) other than methotrexate (MTX), such as iguratimod, salazosulfapyridine (SASP), or tacrolimus (TAC), is unclear. This study investigated the clinical efficacy and retention rates of combinations of ABT and csDMARDs in younger and elderly RA patients over the past 10 years. Additionally, we attempted to elucidate the desirable choice of csDMARDs in combination with ABT and whether these agents could help to sustain high retention rates in daily clinical practice, even in elderly RA patients.

## 2. Materials and Methods

### 2.1. Study Participants, Clinical Investigations and Treatment 

This two-center, retrospective, observational study was performed at Fukushima Medical University School of Medicine, Fukushima, and Ohta Nishinouchi Hospital, Koriyama, Japan. It was approved by the ethics committee of Fukushima Medical University (2019-218). Data on RA patients who received ABT treatment between September 2010 and April 2020 were collected, and their clinical records were retrospectively reviewed. Differences in clinical features (age, sex, disease duration, Steinbrocker stage and classification, tender or swollen joints, and laboratory data), disease activity score 28-joint count C-reactive protein based on three variables (DAS28CRP(3)), therapy (including a past history of previous biologics), complications, and cumulative retention rates were investigated between the elderly and younger RA patients. We also investigated which csDMARDs (MTX, TAC, SASP, and others) were more frequently administered to elderly patients at ABT treatment initiation and which combination of these agents contributed to more sustained retention in RA patients under ABT treatment.

### 2.2. Statistical Analysis

Qualitative data were analyzed using the Fisher exact test. Quantitative data were compared using the Mann–Whitney *U* test or Wilcoxon signed-rank test, based on sample size and distribution. The Kaplan–Meier method was adopted to estimate 2-year or overall retention rates and infection-free survival. A log-rank test was used to analyze the statistical significance between the two groups, and a *p*-value < 0.05 was considered statistically significant. Statistical analyses were performed using Microsoft Excel for Microsoft 365 MSO Add-Ins (Microsoft, Redmond, WA, USA) and Statcel 4 software (OMS Publishing, Saitama, Japan).

## 3. Results

### 3.1. General Characteristics of RA Patients Treated with ABT in This Study 

**Figure 1:**
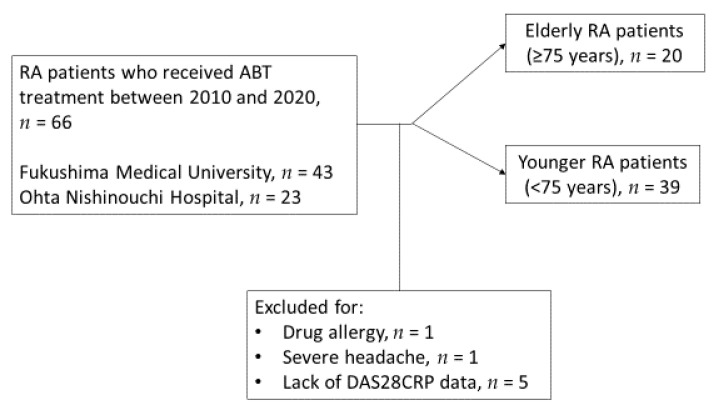
Schema of patients included in this study. Abbreviations: ABT, abatacept; DAS28CRP(3), disease activity score 28-joint count C-reactive protein based on three variables; n, number of patients; RA, rheumatoid arthritis.

Of the 66 patients who received ABT, 59 were included in the final study cohort (Figure 1). The mean age of ABT initiation in our study cohort was 67.5 years old, and the mean disease duration of RA was almost 10 years (12.6 months). More than half of patients showed Steinbrocker stage 3 or higher. The mean DAS28CRP(3) levels were 3.16 at ABT initiation. Among RA patients treated with ABT, 93.2% (55/59) were positive for rheumatoid factor (RF) and/or anti-cyclic citrullinated peptide (CCP) antibodies (seropositive RA) (Table 1).

### 3.2. Clinical Features Complications and the Choice of Concomitant Therapy in Elderly RA Patients Treated with ABT

We compared the clinical features, treatment, and complications in elderly (aged ≥75 years) and younger (aged <75 years) RA patients treated with ABT in our cohort (Table 2). The clinical features in elderly RA patients were similar compared to younger RA patients, except for a lower amount of total tender joints and an increased frequency of Steinbrocker Class 3 or higher. In treatment, the combination of ABT and DMARDs was quite different between the two groups. Compared with the younger RA patients, the elderly RA patients received more TAC (50.0% vs. 12.8%) and SASP (40.0% vs. 10.3%) and less MTX (15.0% vs. 53.8%). Additionally, the time of ABT administration in the course of the disease was considered late (ABT only received after ≥3 other biologics had been used) in a smaller number of elderly RA patients than younger RA patients (5.0% vs. 30.8%). Elderly patients had an increased frequency of malignancies, including prostate cancer, myelodysplastic syndrome, intraductal papillary mucinous neoplasm, and squamous cell carcinoma of the lower leg, and they were all able to resume ABT treatment under careful observation and surgical treatment. However, a younger RA patient had to cease ABT treatment because of lung cancer development. Infections requiring rehospitalization or temporary cessation of treatment were observed in both groups, and the most common infection was pneumonia. The most common reason for ABT cessation was treatment inefficacy. Infections and malignancy were also important causes of ABT cessation.

### 3.3. Clinical Efficacy, Treatment and Overall Retention Rates of ABT in Elderly RA Patients 

**Figure 2:**
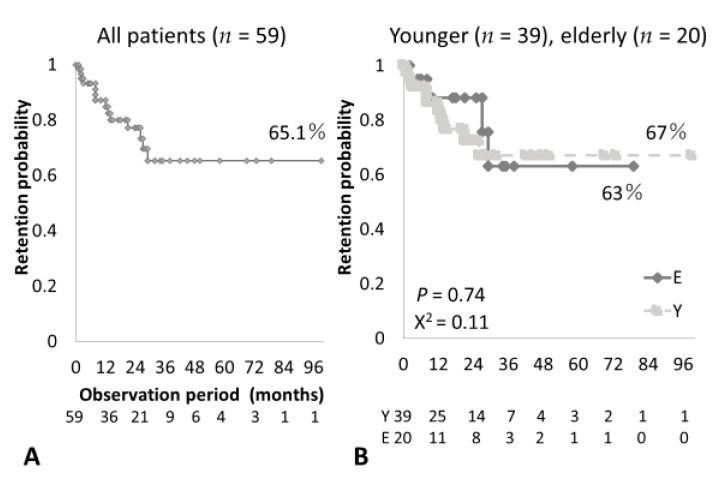
Cumulative retention rates in rheumatoid arthritis (RA) patients who received abatacept (ABT) treatment in this study. (**A**) The overall retention rate in all RA patients who received ABT was 65.1%. (**B**) Comparison of retention rates between younger (aged <75 years) and elderly (aged ≥75 years) RA patients, indicating similar retention rates between the two groups.

The efficacy of ABT (at 6 months) seemed good in elderly as well as younger RA patients. DAS28CRP(3) levels were successfully significantly decreased in both groups (Supplementary Figure S1A). European League Against Rheumatism responses at 6 months of treatment were similar between the two groups (Supplementary Figure S1B). The overall ABT retention rates were 65.1% in our cohort (Figure 2A). Notably, elderly patients showed sustained overall retention rates similar to those observed in younger patients (63% and 67%, respectively; Figure 2B). 

**Figure 3:**
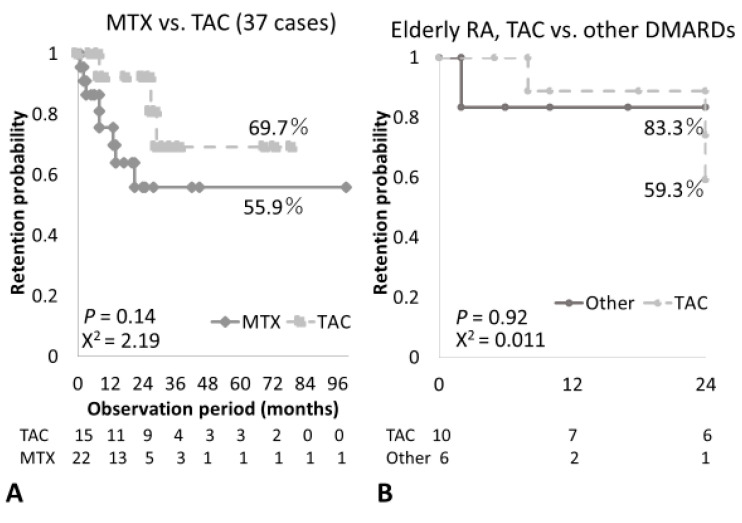
A comparison of retention rates between rheumatoid arthritis patients who received methotrexate (MTX), tacrolimus (TAC), and other disease-modifying antirheumatic drugs (DMARDs) in combination with abatacept. (**A**) A comparison of retention rates between MTX combination (22 cases) and TAC combination (15 cases) in all patients. (**B**) A comparison of retention rates in elderly RA patients between TAC combination and other DMARD combinations.

Next, we investigated which DMARDs contributed to sustained remission in combination therapy with ABT. ABT/TAC showed a relatively higher retention rate than ABT/MTX, but this was not significant (69.7% vs. 55.9%, *p* = 0.14; Figure 3A). Patients who received ABT/TAC were significantly older than those who received ABT/MTX (75.1 years old vs. 62 years old, *p* < 0.01) and showed relatively longer drug retention (31.8 months vs. 19.7 months, *p* = 0.054; Table 3). However, ABT/TAC combination therapy did not show significantly higher 2-year retention rates compared with other DMARDs, including MTX (Figure 3B) in elderly RA patients. Infection-free survival was slightly lower in elderly patients but not significant when compared with younger patients (72.6% vs. 88.4%) (Supplementary Figure S2A). Most RA patients had anti-CCP and/or RF (93.2%, Table 1), and a very small number of seronegative RA patients (RF- and anti-CCP-negative) were observed without a decrease in overall retention rates (Supplementary Figure S2B).

## 4. Discussion

Our study showed that the mean age at ABT initiation in our cohort was 67.5 years, which was relatively older than another recent Japanese cohort study (64.4 years, ANSWER cohort) [7]. The overall retention rates of ABT and infection-free survival, respectively, were similar in the younger and elderly RA patients, indicating that ABT treatment is a good choice to obtain sustained remission in elderly RA patients in daily clinical practice, even if they are aged ≥75 years. Previously, Lahaye et al. have reported that ABT was effective even in RA patients with >75 years in the French cohort (ORA registry), and the risk of severe infection should be considered in such patients [3]. A Belgian cohort study (sub-analysis of the ACTION study) also showed sustained long-term retention rates (up to 5-year retention) in RA patients treated with ABT [9]. ABT treatment has been reported to have relatively higher retention rates than other biologics [6,7,8]. A recent report has described that ABT treatment in elderly RA patients (≥75 years) had better 3-year retention rates than TNF inhibitors (FIRST registry) [8]. However, several csDMARDs (not only MTX) are used in combination with ABT in daily clinical practice. Our study showed that elderly patients tended to receive ABT in combination with TAC or SASP rather than MTX. The differences in the choice of csDMARDs between the two patient groups were attributed to the following factors: ABT treatment alone can be sufficient without MTX combination [10,11], the risk of severe infection in elderly patients receiving ABT is relatively low [5], and, importantly, adverse events are a concern in elderly RA patients treated with MTX. This increased risk of adverse events in MTX treatment may be due to an increased decline of organ functions, including the kidneys, in elderly RA patients [2,12,13]. Furthermore, MTX may initiate some fatal adverse events, such as interstitial lung disease (ILD), and ILD is also a risk factor for poor prognosis (nevertheless, the influence of MTX in RA-ILD development is still controversial: a recent report has described that MTX use can be protective for the development of RA-ILD) [14,15]. In contrast, recent reports have described ABT treatment as relatively safe, and it can be a good option in RA-ILD patients [16,17]. For instance, a Spanish multicenter study revealed using high-resolution computed tomography (HRCT) that 50% of RA-ILD patients was stable after 1 year of ABT initiation. Furthermore, 36% of RA-ILD patients showed improved HRCT findings [17]. Bone marrow suppression is also a critical adverse event in MTX-treated RA patients [11]. On the other hand, lymphopenia (<1000/µL) itself showed an increased risk of infections during ABT treatment in post-marketing surveillance of ABT in Japan [5], even though cytopenia itself is rarely observed in ABT treatment [18,19]. Hematologic malignancies, especially MTX-associated lymphoproliferative disorder (MTX-LPD), are also important complications in MTX treatment. In fact, our cohort included two patients with a history of MTX-LPD (one patient needed chemotherapy). These patients received the csDMARDs bucillamine and SASP upon ABT initiation.

Undeniably, when using biologics for RA patients, combination treatment with DMARDs is a crucial issue. Most biologics, including ABT, have been used with MTX for combination therapy [20]. However, reports regarding the use of TAC in combination with biologics are limited [21,22,23]. In daily clinical practice, a substantial number of RA patients are unable to use MTX due to intolerance and/or its adverse events as combination therapy with biologics. In Japan, TAC was approved for RA treatment in 2005 and is a routinely used DMARD. TAC inhibits calcineurin activity in T cells by binding to an FK506-binding protein, which results in the suppression of various cytokines, including interleukin (IL)-2, interferon-γ, and tumor necrosis factor (TNF)-α [24]. A past report has demonstrated that TAC treatment was effective for active RA patients at a dosage of 1.5–3 mg/day without serious safety concerns [25]. Kawai et al. also have indicated that TAC is effective and relatively safe even in elderly RA patients who had an insufficient response to DMARDs [24]. High retention rates of TAC treatment were also reported in South Korean RA patients with a favorable efficacy and safety profile (69.1% of 4-year retention rates) [26]. In fact, relatively higher retention rates were observed in our cohort (69.7%) in ABT/TAC combination group. Thus, some Japanese doctors use TAC as an alternative to MTX, even if ABT monotherapy is sufficiently effective in certain patients. Ishida et al. and Takeuchi et al. evaluated the safety and effectiveness of add-on TAC in Japanese patients with RA who had an inadequate response to biologics. [22,27], revealing that TAC was good enough and well-tolerated in Japanese patients who were resistant to biologics. Furthermore, some Japanese authors have described the real-world effect of ABT/TAC combination therapy [28,29]. Fujibayashi et al. reported that ABT/TAC, as well as ABT/MTX combination therapy, was effective in Japanese RA patients [28]. Suzuki et al. also reported that additional administration of TAC in RA patients who showed an inadequate response to ABT was effective [29]. Our study also showed that ABT/TAC combination therapy was effective in elderly as well as younger RA patients, leading to sustained remission rates and implicating a profound combination effect of ABT with TAC. ABT blocks CD28-mediated co-stimulatory signals in antigen presentation to T cells, resulting in reduced CD4 T cells and inflammatory cytokines (IL-2 and TNF-α). TAC also reduces cytokine production by inhibiting calcineurin dephosphorylation in T cells [30,31]. These two distinct mechanisms of T cell and cytokine regulation may enhance the therapeutic effect and sustained retention in combination therapy [28]. Collectively, in consideration of sustained retention rates, ABT/TAC can be a good choice in the treatment of elderly RA patients, especially those who are intolerant to MTX.

In our study, there were no significant differences in complications between younger and elderly RA patients, including the frequency of osteoporotic fracture, cardio/cerebrovascular disease, ILD, infections and the reason for cessation (inefficacy was the most frequent reason), with the exception of the occurrence of neoplasms. ABT has been reported as only associated with an increased risk of melanoma and, generally, no significant cancer risk was noted in real-world data compared with other biologics or Janus kinase inhibitors [32,33]. In our study, no particular cancer type was observed (Table 2); nevertheless, careful monitoring of malignancies should be performed. In fact, a younger patient in our cohort had to cease ABT treatment for lung cancer, and he underwent lung surgery for curative treatment.

This study had several limitations. The study design was retrospective, observational, and comprised a relatively small number of patients in 2 regional centers. Because of its retrospective nature, a distinct evaluation of RA activity, including DAS28ESR, the Simplified Disease Activity Index, and the Clinical Disease Activity Index, was not performed. A radiologic evaluation was also difficult because the timing of the radiologic images was not the same (or not taken) for each patient. Furthermore, because we did not routinely measure serum TAC levels, we might have missed adverse events caused by a TAC overdose. Nevertheless, we demonstrated that ABT/TAC combination therapy could be a good choice in elderly RA patients with regard to better retention rates as well as in younger patients in daily clinical practice.

## 5. Conclusions

ABT treatment is effective in elderly (aged ≥ 75 years) as well as younger (aged < 75 years) RA patients. Combination therapy of ABT/TAC and ABT/SASP may contribute to sustained retention rates in elderly RA patients, indicating TAC can be a good choice in elderly RA patients when receiving ABT treatment. Further large-scale, double-blind, randomized studies are necessary to clarify the precise effect of TAC combination therapy for sustaining drug retention rates in elderly patients with RA receiving ABT.

## Figures and Tables

**Table 1:** General clinical characteristics in patients with rheumatoid arthritis treated with abatacept in our study.

General Characteristics (*n* = 59)	Average ± Standard Deviation
Age (years) Male/female (number), female (%)	67.5 ± 12.6 20/39 (66.1)
Disease duration (months)	126 ± 133
Steinbrocker Stage (III/IV)	33/59 (55.9)
Steinbrocker Class (III/IV)	9/59 (15.3)
Swollen joint count	3.03 ± 3.58
Tender joint count	3.22 ± 4.44
CRP (mg/dL)	2.01 ± 2.22
ESR (mm/h)	37.4 ± 27.2
RF- or anti-CCP antibody-positive (%)	55/59 (93.2)
Methotrexate, *n* (%), (mean mg/week)	24/59 (40.7%), 7.08 mg/week
Tacrolimus. *n* (%), (mean mg/day)	15/59 (25.4%), 1.6 mg/day
Observation period in months (range)	23.1 ± 20.7 months (1–99)

The results are shown as mean ± standard deviation or number (%), unless otherwise indicated. CCP, cyclic citrullinated peptide; CRP, C-reactive protein; ESR, erythrocyte sedimentation rate; RF, rheumatoid factor.

**Table 2:** Clinical features, treatment, and complications in rheumatoid arthritis patients treated with abatacept in our cohort.

Clinical Items	Elderly (75≤)	Younger (75>)	*p*
Number	20	39	-
Age (years) Male/female (female, %)	78.4 ± 2.56 4/16 (80%)	62.0 ± 12.1 8/31 (79.5%)	- NS
Disease duration (month)	149 ± 135	114 ± 132	NS
Steinbrocker Stage (III/IV) Class (III/IV)	14/20 (70%) 6/20 (30%)	19/39 (48.7%) 3/39 (7.7%)	NS 0.03
Swollen joint count (SJC)	1.8 ± 1.58	3.67 ± 4.14	NS
Tender joint count (TJC)	1.5 ± 2.26	4.1 ± 5.02	0.02
CRP (mg/dL)	1.83 ± 2.07	2.1 ± 2.32	NS
ESR (mm/1hour)	41.4 ± 31.1	35.2 ± 25.0	NS
DAS28CRP(3)	2.79 ± 0.71	3.35± 1.15	NS
Retention period (months)	21.6± 20.2	23.9± 21.2	NS
Treatment			
Methotrexate (%)	3/20 (15.0)	21/39 (53.8)	<0.01
Tacrolimus	10/20 (50.0)	5/39 (16.7)	<0.01
Salazosulfapyridine	8/20 (40.0)	4/39 (10.3)	0.01
Prednisolone Doses (mg/day)	12/20 (60.0) 6.71± 2.58	24/39 (61.5) 4.98± 2.78	NS NS
Timing of ABT use for 1st Biologics (%)	8/20 (40.0)	15/39 (38.5)	NS
2nd Biologics (%)	11/20 (55.0)	12/39 (29.7)	NS
3rd or more (%)	1/20 (5.0)	12/39 (33.3)	0.02
Complication			
Interstitial pneumonia	5/20 (25%)	6/39 (15.4%)	NS
Osteoporotic fracture	2/20 (10%)	4/37 (10.2%)	NS
Cardio/cerebrovascular disease	3/20 (15%)	2/39 (5.1%)	NS
Malignancy	4/20 (20%) Prostate cancer, Myelodysplastic syndrome, IPMN SCC (lower leg)	1/39 (2.6%) Lung cancer	0.04
Infections	3/20 (15%) Pyelonephritis Pneumonia 2	4/39 (10.3%) Phlegmon 2 Pneumonia, Lung abscess VZV infection	NS
ABT cessation Reason	4/20 (20%) Ineffective 2 Infection 1 Brain hemorrhage 1	9/39 (23.1%) Ineffective 6 Lung cancer 1 Kidney dysfunction 1 Infection 1	NS

The results are shown as mean ± standard deviation or number (%), unless otherwise indicated. Abbreviations: ABT, abatacept; CRP, C-reactive protein; ESR, erythrocyte sedimentation rate; DAS28CRP(3), disease activity score 28-joint count C-reactive protein based on three variables; IPMN, intraductal papillary mucinous neoplasm; NS, not significant; SCC, squamous cell carcinoma; VZV, varicella-zoster virus.

**Table 3:** A comparison of clinical characteristics between abatacept/tacrolimus and abatacept/methotrexate combination treatments in patients with rheumatoid arthritis.

Clinical Characteristics	Abatacept/Tacrolimus	Abatacept/Methotrexate	*p*-Value
Number of patients treated	15	22	-
Age (years) Male/female (female, %)	75.1 ± 7.93 2/13 (86.7)	62.0 ± 14.8 6/16 (72.7)	<0.01 NS
Disease duration (month)	140 ± 121	114 ± 136	NS
Steinbrocker Stage (III/IV)	12/15 (80.0)	12/22 (54.5)	NS
Steinbrocker Class (III/IV)	2/15 (13.3%)	2/22 (9.1%)	NS
Retention period (months)	31.8 ± 24.2	19.7 ± 25.0	0.054
DAS28CRP(3)			
Before treatment	3.27 ± 1.33	3.31 ± 1.13	NS
Six months after treatment	2.16 ± 0.78	2.26 ± 0.92	NS

The results are shown as mean ± standard deviation or number (%), unless otherwise indicated. DAS28CRP(3), disease activity score 28-joint count C-reactive protein based on three variables; NS, not significant.

## Data Availability

The data presented in this study are available on request from the corresponding author. The data are not publicly available due to the information that could compromise the privacy of research participants.

## Supplementary Materials

The following are available online at https://www.mdpi.com/article/10.3390/medicina57090914/s1. Figure S1: Clinical efficacy of abatacept at 6 months of treatment. A. DAS28CRP(3) levels were significantly decreased in younger (aged <75 years) and elderly (aged ≥75 years) rheumatoid arthritis patients. B. EULAR response after 6 months of ABT treatment between the two groups. *p*-values < 0.05 were considered statistically significant. ABT, abatacept; DAS28CRP(3), disease activity score 28-joint count C-reactive protein based on three variables; EULAR, European League Against Rheumatism, m, months; NS, not significant, Figure S2: Comparison of infection-free survival in younger/elderly patients and overall retention rates in seropositive RA patients. A. Cumulative overall infection-free survival rates between elderly and younger patients. B. Overall retention rates between seropositive (anti-cyclic citrullinated peptide [CCP]-positive or rheumatoid factor [RF]-positive) and seronegative (negative for both anti-CCP antibody and RF) RA patients. E, elderly patients (≥75 years); neg, negative; pos, positive; Y, younger patients (<75 years).

## Abbreviations

ABT, abatacept; CCP, cyclic citrullinated peptide; CD; cluster of differentiation; CRP, C-reactive protein; csDMARDs, conventional synthetic disease-modifying antirheumatic drugs; DMARDs, disease-modifying antirheumatic drugs; ESR, erythrocyte sedimentation rate; DAS28CRP(3), disease activity score 28-joint count C-reactive protein based on three variables; HRCT, high-resolution computed tomography; ILD, interstitial lung disease; IL, interleukin; IPMN, intraductal papillary mucinous neoplasm; MTX, methotrexate; NS, not significant; RA, rheumatoid arthritis; RF, rheumatoid factor; SASP, salazosulfapyridine; SCC, squamous cell carcinoma; TAC; tacrolimus; TNF; tumor necrosis factor; VZV, varicella-zoster virus.
