# Supplementary material for: Sustained Long-Term Retention Rates of Abatacept in Combination with Conventional Synthetic Disease-Modifying Antirheumatic Drugs in Elderly Patients with Rheumatoid Arthritis
Source: Medicina (Kaunas). 2021 Aug 31;57(9):914. doi: 10.3390/medicina57090914 (PMC8469009; doi:10.3390/medicina57090914)
Supplement: Supplementary file 1 [file medicina-57-00914-s001.zip › medicina-1315618-supplementary.pdf]

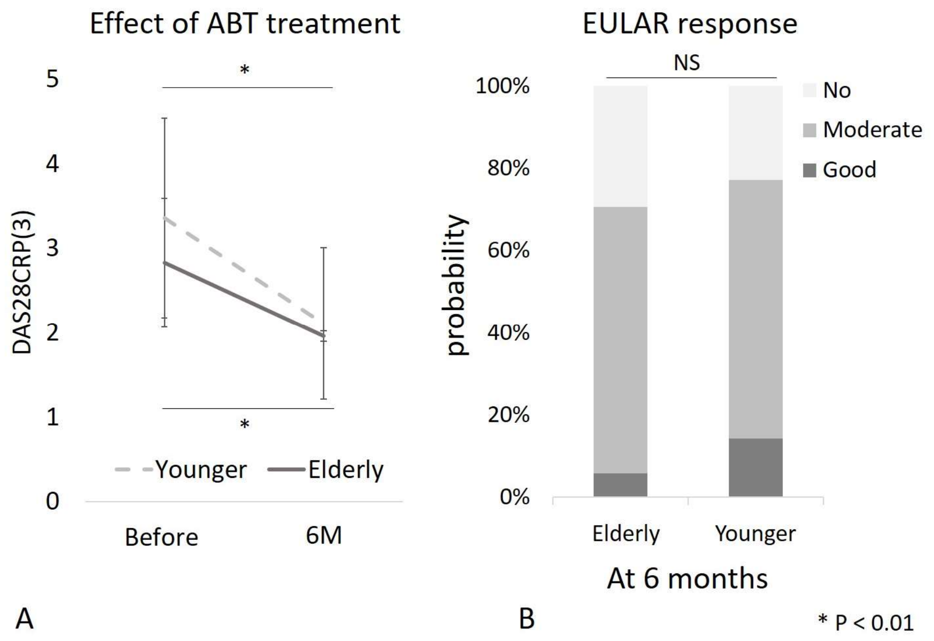

Supplementary Figure S1. Clinical efficacy of abatacept at 6 months of treatment. **A**. DAS28CRP(3) levels were significantly decreased in younger (aged <75 years) and elderly (aged ≥75 years) rheumatoid arthritis patients. **B**. EULAR response after 6 months of ABT treatment between the two groups. *p*-values < 0.05 were considered statistically significant. ABT, abatacept; DAS28CRP(3), disease activity score 28-joint count C-reactive protein based on three variables; EULAR, European League Against Rheumatism, m, months; NS, not significant.

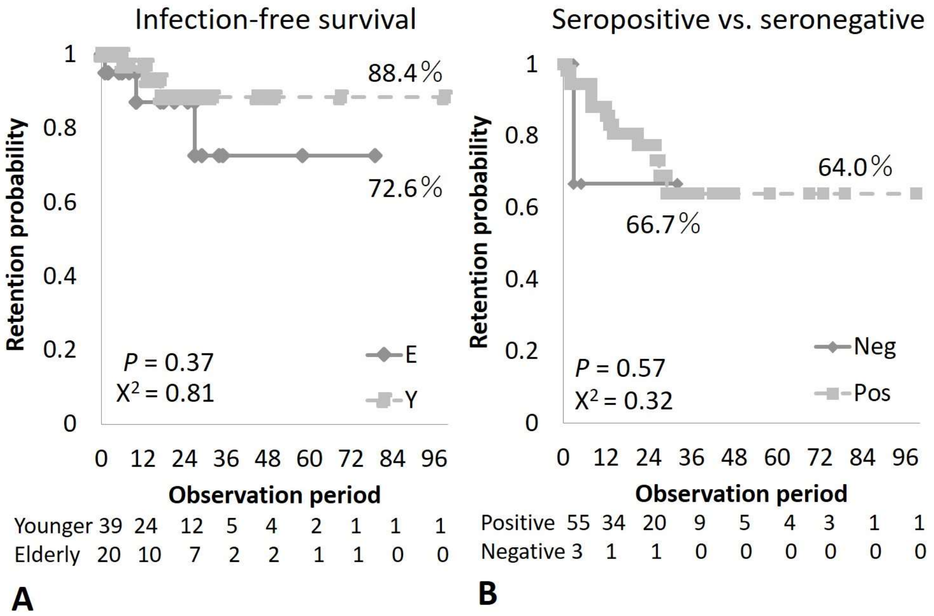

Supplementary Figure S2. A comparison of infection-free survival in younger/elderly patients and overall retention rates in seropositive RA patients. A. Cumulative overall infection-free survival rates between elderly and younger patients. **B**. The overall retention rates between seropositive (anti-cyclic citrullinated peptide [CCP]-positive or rheumatoid factor [RF]-positive) and seronegative (negative for both anti-CCP antibody and RF) RA patients. E, elderly patients (≥75 years); neg, negative; pos, positive; Y, younger patients (<75 years).
